# Influence of Concurrent Finger Movements on Transcranial Direct Current Stimulation (tDCS)-Induced Aftereffects

**DOI:** 10.3389/fnbeh.2017.00169

**Published:** 2017-09-12

**Authors:** Yuichiro Shirota, Daniella Terney, Andrea Antal, Walter Paulus

**Affiliations:** Department of Clinical Neurophysiology, University Medical Center Göttingen, Georg-August University Göttingen, Germany

**Keywords:** muscle activation, motor evoked potential, transcranial direct current stimulation, transcranial magnetic stimulation

## Abstract

Transcranial direct current stimulation (tDCS) has been reported to have bidirectional influence on the amplitude of motor-evoked potentials (MEPs) in resting participants in a polarity-specific manner: anodal tDCS increased and cathodal tDCS decreased them. More recently, the effects of tDCS have been shown to depend on a number of additional factors. We investigated whether a small variety of movements involving target and non-target muscles could differentially modify the efficacy of tDCS. MEPs were elicited from the right first dorsal interosseous muscle, defined as the target muscle, by single pulse transcranial magnetic stimulation (TMS) over the primary motor cortex (M1). During M1 tDCS, which lasted for 10 min applying anodal, cathodal, or sham condition, the participants were instructed to squeeze a ball with their right hand (Task 1), to move their right index finger only in the medial (Task 2), in the lateral direction (Task 3), or in medial and lateral direction alternatively (Task 4). Anodal tDCS reduced MEP amplitudes measured in Task 1 and Task 2, but to a lesser extent in the latter. In Task 3, anodal tDCS led to greater MEP amplitudes than cathodal stimulation. Alternating movements resulted in no effect of tDCS on MEP amplitude (Task 4). The results are congruent with the current notion that the aftereffects of tDCS are highly variable relying on a number of factors including the type of movements executed during stimulation.

## Introduction

Transcranial direct current stimulation (tDCS) was originally introduced as a powerful tool to modulate cortical excitability bidirectionally, depending on the polarity with which it was applied. The first studies reported that anodal tDCS increased the amplitude of the motor evoked potentials (MEPs), probably by increasing the excitability of the primary motor cortex (M1) (Nitsche and Paulus, 2000, 2001) while cathodal tDCS diminished it (Nitsche and Paulus, 2000; Nitsche et al., 2003). The anodal-excitatory and cathodal-inhibitory association was reproduced in later studies (for review, see Nitsche and Paulus, 2001; Nitsche et al., 2003, 2008). Pharmacological evidence suggested that altered membrane potential and synaptic plasticity contributed to the polarity-specific aftereffects of tDCS (Liebetanz et al., 2002; Nitsche et al., 2003a). The positive influence of tDCS on learning (Nitsche et al., 2003b; Galea and Celnik, 2009; Reis et al., 2009) promoted its therapeutic applications, including those for stroke rehabilitation (Kang et al., 2016; Meinzer et al., 2016).

Recently, however, the notion of constant, polarity-specific bidirectional aftereffects has met considerable challenges. Non-invasive brain stimulation in general is subject to a number of determinants (Ridding and Ziemann, 2010), and tDCS is not an exception. The aftereffects of tDCS can be altered by such factors as stimulation parameters (Batsikadze et al., 2013; Monte-Silva et al., 2013), concurrent peripheral stimulation (Schabrun et al., 2013; Rizzo et al., 2014), or activities of the participants during (Antal et al., 2007; Bortoletto et al., 2015) or after (Thirugnanasambandam et al., 2011) stimulation. Furthermore, they can differ simply due to the inter-individual variability among participants (López-Alonso et al., 2014; Wiethoff et al., 2014; Chew et al., 2015; Strube et al., 2016), for which anatomical, physiological, genetic and other characteristics of the individuals have been proposed to play different roles (Antal et al., 2010; Wiethoff et al., 2014; Laakso et al., 2015; Opitz et al., 2015). Because of the increasing use of this technique in research as well as for clinical purposes it is important to determine the sources or predictors of such variability (see e.g., Nuzum et al., 2016) that can alter the aftereffects in a systematic way (Brunoni et al., 2012; Hashemirad et al., 2016; Kang et al., 2016).

In this study, we investigated the influence of various motor tasks on concurrent tDCS, as potential factors affecting the polarity-specific efficacy of tDCS. These were chosen to allow confirmation of previous findings (Antal et al., 2007). Among various stimulation parameters, polarity can play a key role in defining both direction and magnitude of tDCS effects on neurons (Rahman et al., 2013; Lafon et al., 2017); thus we also compared three different tDCS conditions (i.e., anodal, cathodal, and sham) for each of the motor tasks.

## Methods

### Participants

Twenty eight healthy volunteers (16 male, all right-handed) were recruited for the study which conformed to the Declaration of Helsinki, and was approved by the Ethics Committee of the University of Göttingen. Inclusion criteria were an age between 20 and 30 years, right-handedness according to the Edinburgh Handedness Inventory (Oldfield, 1971), and written informed consent. Exclusion criteria were any neurological or psychological disorder, metallic implants or implanted electric devices, or any regular medication.

The participants were randomly allocated to perform predefined tasks, which are described below. Each task group consisted of 12 participants; 15 participants performed only one task, seven took part in two, five in three, while one participated in all four tasks.

### Transcranial direct current stimulation (tDCS)

Direct current was delivered by a battery-driven, constant-current stimulator (NeuroConn GmbH, Ilmenau, Germany) through conductive-rubber electrodes (5 × 7 cm) encased in saline-soaked sponges. One of the electrodes was placed over the representational field of the right first dorsal interosseous muscle (FDI) as identified by TMS (see below), while the other electrode was located contralaterally above the right eyebrow. The type of stimulation (anodal or cathodal) refers to the polarity of the electrode above the M1. The current was applied for 10 min with an intensity of 1.0 mA. The fade-in/fade-out time was 8 s. For sham stimulation the current was turned on for 15 s at the beginning of the stimulation in order to achieve the slight itching sensation under the electrode. Participants were blinded for stimulation conditions in all of the experiments.

### Transcranial magnetic stimulation (TMS)

To detect changes in motor cortical excitability, MEPs of the right FDI were recorded following stimulation of its motor-cortical representational field by single-pulse TMS delivered by a Magstim 200^2^ stimulator (Magstim Company, Whiteland, Wales, UK) and a figure-of-eight standard double magnetic coil (diameter of one winding, 70 mm). The coil was held tangentially to the skull, with the handle pointing posteriorly and laterally at approximately 45° from the midline, resulting in a posterior–anterior direction of current flow in the brain. The optimal position was defined as the site where stimulation consistently evoked the largest MEPs. The site was marked with a skin marker to ensure that the coil was held in the correct position throughout the experimental session. Surface electromyography (EMG) was recorded from the right FDI using a pair of Ag/AgCl electrodes in a belly-tendon montage. The signals were amplified and filtered (2 Hz–3 kHz), digitized at five kHz with a micro 1401 AD converter (Cambridge Electronic Design, Cambridge, UK), and stored using Signal software (Cambridge Electronic Design, version 2.13). Data were analyzed offline on a personal computer. Complete muscle relaxation was achieved using visual feedback of the EMG activity. The intensity of the stimulator output was adjusted at the baseline recording so that the stimulus led to an MEP with an average peak-to-peak amplitude of approximately one mV (SI1mV).

### Experimental sessions

#### Tasks

**Figure 1:**
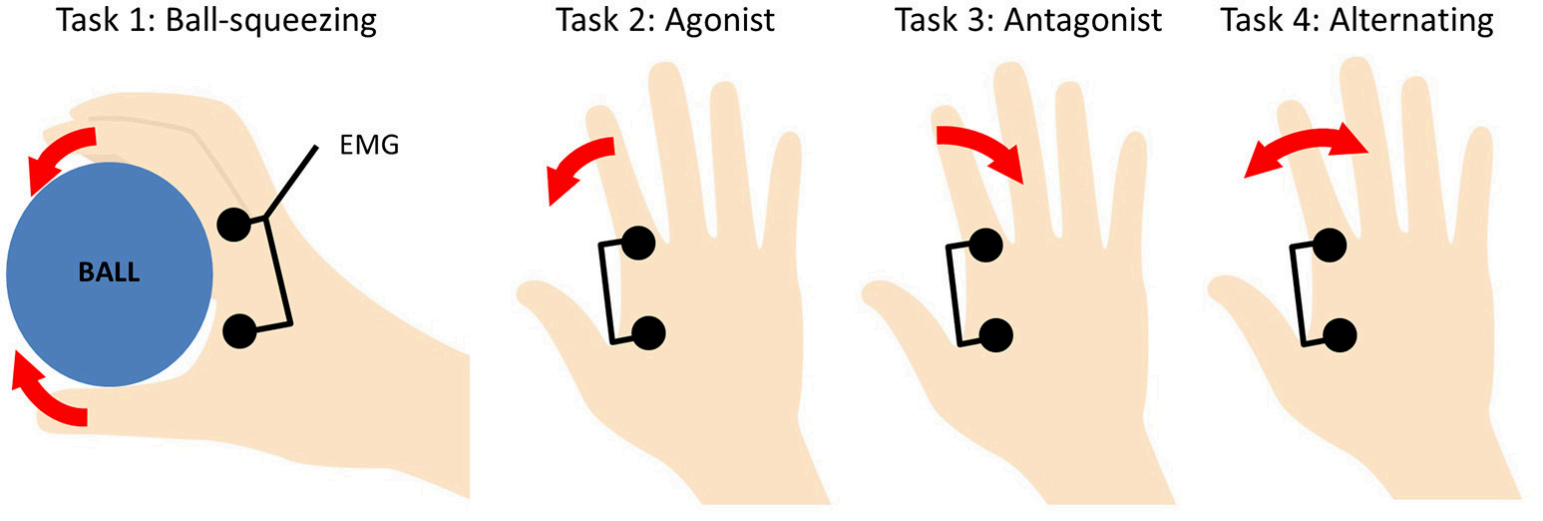
Schematic description of the tasks. In Task 1, a ball with 8-cm diameter (shown in blue) was squeezed with the entire hand. In the other tasks, the right index finger was moved in a specified direction (Tasks 2–4).

The participants were instructed to perform the following tasks: squeeze an 8 cm-diameter ball with their right hand to half-maximal contraction (Task 1; Antal et al., 2007), move their right index finger either in the medial-agonist direction (Task 2), or in the lateral-antagonist direction (Task 3), or move it alternately in the medial and lateral direction (Task 4). Figure 1 shows each of the tasks schematically. The finger was to be moved at a frequency of 1 Hz. The task performance during the tDCS was continuously monitored by the experimenter through visual inspection to check whether the movements of the participant were compatible with the task assigned in that session. If it was necessary, the participants were reminded to keep the magnitude of movement as stable as possible. The stimulation mode for each task session was randomly assigned. Only one task was performed during each session with at least a 4-day interval between sessions. For participants performing more than one task, each series of three sessions was completed before the next task series began. During the sessions the participants were seated in a reclining chair.

#### Stimulation

After having identified the motor-cortical representational field of the right FDI the resting and active motor thresholds (RMT and AMT) were determined using the relative frequency method (Rossini et al., 2015). The participants then relaxed for at least 5 min after which period the baseline was determined by recording 25 MEPs using a stimulus intensity of SI1mV at an inter-stimulus interval of 4.0 ± 0.4 s. The tDCS electrodes were then placed as described above.

At the end of stimulation with task performance, the participants were allowed to relax for 5 min which previous studies had shown to be necessary for adequate relaxation (Antal et al., 2007; Terney et al., 2008). Following the rest, 25 MEPs were recorded every 5 min for the first 30 min and then every 15 min for the next 30 min.

### Statistical analysis

The RMT, AMT, SI1mV, and baseline MEP amplitudes were compared between the stimulation conditions using one-way analysis of variance (ANOVA), separately for each task.

As the index for the main analysis, mean MEP amplitude was calculated for each of the time bins covering baseline and post-stimulation values. Then, mean post-tDCS MEP amplitudes were individually normalized to the baseline value. An ANOVA model for repeated measures was calculated with the normalized MEP amplitude as the dependent variable, TASK as a between-subjects factor, and STIM (anodal, cathodal, and sham) and TIME (5, 10, 15, 20, 25, 30, 45, and 60 min after tDCS) as within-subject factors. We chose this to obtain greater power in determining the effects of polarity on direction and magnitude of tDCS effects (Rahman et al., 2013; Lafon et al., 2017). If a significant interaction was found, we further performed the analysis of simple main effects with the Bonferroni correction for multiple comparisons. Effects were considered significant if *p* < 0.05.

## Results

All of the participants tolerated tDCS, and none had severe adverse-effects during or after the stimulation. One subject in the Task 2 group reported a strong, uncomfortable skin sensation during anodal stimulation, and the stimulation had to be discontinued after 7 min. RMT, AMT, SI1mV, and baseline MEP amplitudes were compared between anodal, cathodal and sham conditions within each task. There were no significant differences except for the RMT in Task 4 (see Table 1).

**Table 1:** Baseline characteristics.

	**Anodal**	**Cathodal**	**Sham**	***F*-value**	***p*-value**
**RMT (%MSO)**					
Task 1	37.9 ± 4.87	40.8 ± 6.51	38.1 ± 4.66	1.100	0.345
Task 2	37.6 ± 7.01	40.0 ± 8.32	39.5 ± 7.23	0.283	0.755
Task 3	37.9 ± 6.40	39.6 ± 8.15	37.8 ± 6.89	0.227	0.798
Task 4	38.4 ± 2.54	41.6 ± 4.10	38.3 ± 2.39	*4.268*	0.022^*^
**AMT (%MSO)**					
Task 1	31.2 ± 3.30	32.9 ± 5.96	31.5 ± 2.81	0.572	0.570
Task 2	29.4 ± 4.56	31.0 ± 6.38	30.8 ± 6.55	0.281	0.772
Task 3	30.0 ± 5.70	30.4 ± 6.84	29.3 ± 5.38	0.099	0.906
Task 4	31.5 ± 2.68	33.4 ± 4.31	31.3 ± 2.05	1.685	0.201
**SI1mV (%MSO)**					
Task 1	50.1 ± 9.38	52.9 ± 9.23	50.3 ± 9.47	0.346	0.710
Task 2	47.9 ± 10.4	49.9 ± 11.6	49.9 ± 11.0	0.132	0.877
Task 3	48.3 ± 10.4	48.8 ± 10.8	47.6 ± 10.1	0.038	0.962
Task 4	49.8 ± 5.04	53.1 ± 5.68	50.2 ± 4.72	1.438	0.252
**Baseline MEP (mV)**					
Task 1	0.95 ± 0.11	0.94 ± 0.15	1.04 ± 0.16	2.044	0.146
Task 2	0.92 ± 0.17	0.99 ± 0.14	1.00 ± 0.13	0.865	0.430
Task 3	0.98 ± 0.17	0.97 ± 0.14	1.01 ± 0.11	0.256	0.776
Task 4	0.98 ± 0.13	0.95 ± 0.08	1.03 ± 0.15	1.291	0.289

*Values are given as mean ± standard deviation. Each Task condition contains different set of participants. Task 1, ball-squeezing; Task 2, agonist movements; Task 3, antagonist movements; Task 4, alternating movements*.

* *Pair-wise comparison indicated significant difference between cathodal and sham condition (p = 0.045 with the Bonferroni correction)*. The italic value means statistical significance (p < 0.05), as also indicated by the asterisks.

**Figure 2:**
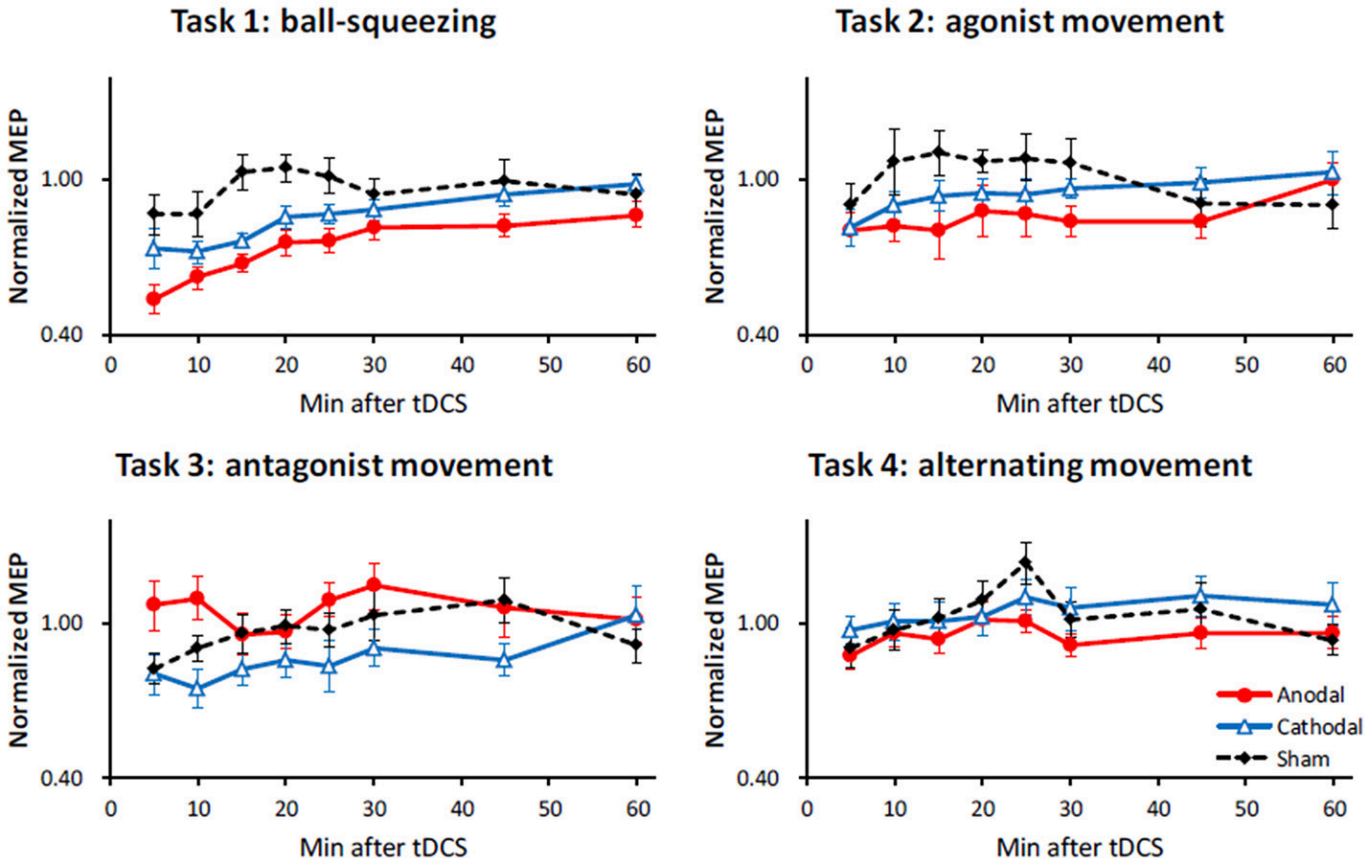
Effect of 10 min anodal, cathodal and sham tDCS on MEP amplitudes with different motor tasks. Each graph shows time course of the normalized MEP amplitude after 10 min of anodal (red filled circles with a solid line), cathodal (blue open triangles with a solid line), and sham (black diamonds with a dashed line) tDCS. The X-axis represents time after tDCS in minutes, and Y-axis the normalized MEP amplitude. Error bars indicate standard error of the mean.

The three-way repeated measures ANOVA revealed a significant main effect of STIM [*F*_(2, 88)_ = 3.92; *p* = 0.023], TIME [*F*_(7, 308)_ = 8.57; *p* < 0.001], and TASK [*F*_(3, 44)_ = 5.32; *p* = 0.003], whereas the three-way interaction was not significant [*F*_(42, 616)_ = 1.01, *p* = 0.45]. More importantly, we found a significant interaction between STIM and TASK [*F*_(6, 88)_ = 3.02; *p* = 0.010], as well as between STIM and TIME [*F*_(14, 616)_ = 2.83; *p* < 0.001]. The significant interaction between STIM and TIME is probably due to the fact that the MEP amplitudes returned to the baseline over the course of 60 min (Figure 2). Given the significant interaction between STIM and TASK (see Figure 2), which implied that each task had a differential impact on different tDCS polarities, we then conducted further analyses of simple main effects. Individual time courses are presented in the Supporting Information (Figure S1).

**Figure 3:**
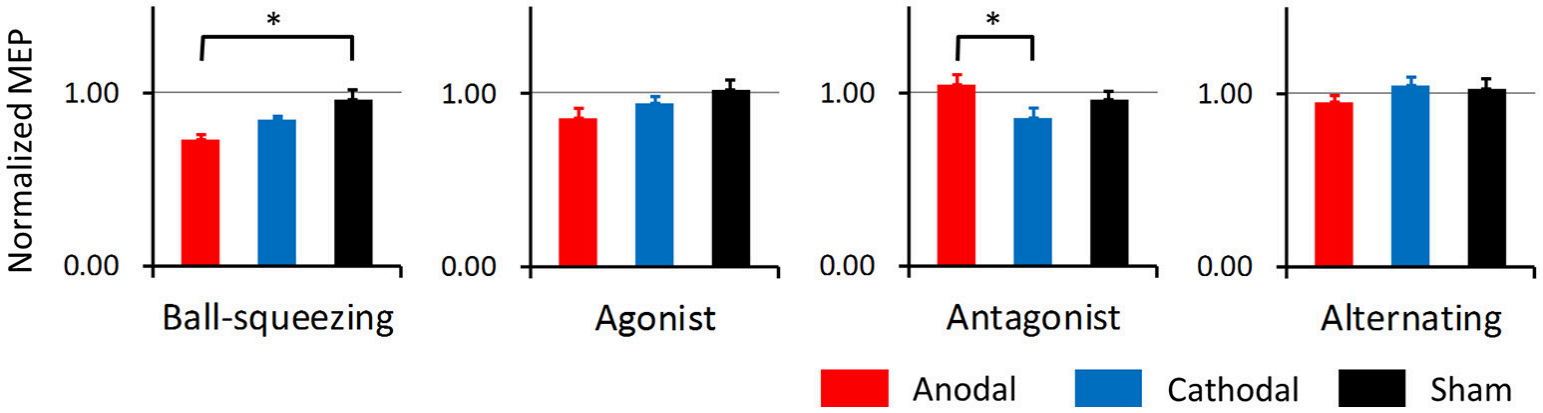
Normalized MEP amplitudes pooled across 60 min after intervention. Based on the time course shown in Figure 2, normalized MEP amplitudes were pooled for the 60 min in each participant and averaged for each task and polarity. As in Figure 1, red bars indicate anodal, blue cathodal, and black sham tDCS. Error bars represent standard errors of the mean. Asterisks denote significant simple main effects in pair-wise comparisons.

The simple main effects of STIM at each level of TASK were calculated using the Bonferroni correction for multiple comparisons. As shown in the pooled data relevant for these analyses (Figure 3), the effect of anodal tDCS on MEP differed significantly from that of sham tDCS in combination with the ball-squeezing task (*p* = 0.013), and results from anodal and cathodal tDCS differed significantly in the antagonist task (*p* = 0.003). Based on these results we further performed a one-way ANOVA where the pooled data for the anodal condition was compared across the four tasks. We found a significant effect of TASK [*F*_(3, 44)_ = 8.26, *p* < 0.001]. A following *post-hoc* test with the Bonferroni correction indicated that the ball-squeezing task resulted in significantly smaller MEPs than the antagonist task (*p* < 0.001) and the alternating task (*p* = 0.011), and that the agonist task resulted in smaller MEPs than the antagonist task (*p* = 0.036). To test whether results of the sham and cathodal tDCS were different among the tasks, additional one-way ANOVAs were performed, revealing a non-significant result [*F*_(3, 44)_ = 0.44, *p* = 0.73] for the sham condition and significance for the cathodal condition [*F*_(3, 44)_ = 4.24, *p* = 0.011]. *Post-hoc* analysis for the latter showed that the normalized MEP amplitude in the alternating task was significantly different from that in the ball-squeezing task (*p* = 0.02 with the Bonferroni correction) and the antagonist task (*p* = 0.033 with the Bonferroni correction), which were probably due to unchanged MEP after the alternating task and decreased MEP after the other two.

## Discussion

In this study we found that different hand and finger movements during tDCS resulted in different modulations of mean MEP amplitudes, indicating that the brain status during tDCS is one of the key factors in determining its effect. Interaction between brain states and type of stimulations has been reported using divers brain stimulation techniques including tDCS (Feurra et al., 2013; Bortoletto et al., 2015). Importantly, the interactions of anodal and cathodal tDCS with the concurrent motor tasks were different, supporting the assumption that polarity is a basic parameter in determining tDCS effects (Rahman et al., 2013; Lafon et al., 2017). Results from the sham stimulation across the tasks, on the other hand, revealed that the motor tasks alone did not significantly increase or decrease the MEP amplitude. Results from the Task 1 (ball-squeezing) confirm previous results, which demonstrated a decrease in the MEP amplitude after anodal tDCS combined with the task (Antal et al., 2007). Some of the movements (i.e., the agonist movements in Task 2) yielded similar results, while others did not.

The effects of tDCS with the agonist movements (Task 2) were similar, albeit non-significant, to those with ball-squeezing where MEP decreased with anodal tDCS. Since the ball-squeezing in Task 1 requires simultaneous activation of the FDI, which is the main effector in Task 2, together with other muscles, this discrepancy between the Task 1 and Task 2 might have stemmed from synergistic recruitment of motor circuits involving FDI (Smith and Fetz, 2009).

Results from Task 3 were in contrast with those from Tasks 1 and 2; the MEP amplitude was greater after anodal tDCS than after cathodal. Such results seem to have something in common with the classical observation of anodal-excitatory and cathodal-inhibitory effects of tDCS (Nitsche and Paulus, 2001; Nitsche et al., 2003, 2008). This resemblance led us to conjecture that a resting target muscle, or a lack of intention to contract it, during tDCS would be one of the prerequisites for the anodal tDCS to increase and cathodal tDCS to decrease MEP amplitude. Depolarization and hyperpolarization of the resting membrane potential have been considered as a key neuronal mechanism for the anodal and cathodal tDCS to have an impact on cortical excitability (Rahman et al., 2013). While simultaneous activation of the target muscle (as in Tasks 1 and 2) obviously disturbs the resting state of it, activation of antagonistic muscles apparently did not, so that the aftereffects of the stimulation might have been more similar to the classical pattern, which could be more explicitly tested by including the resting condition as discussed below. Furthermore, possible modulating effects of concurrent activation of the antagonist muscle should be further investigated.

Interestingly, neither anodal nor cathodal tDCS changed the MEP amplitude when combined with the alternating activation of agonist and antagonist muscles (Task 4). This cannot be simply explained as a summation of the agonist and antagonist activations. Differences in the RMT among tDCS conditions might have been related to the results, but we believe that such an effect, if any, was marginal, given the non-significance in the other baseline values including SI1mV and MEP amplitude (see Table 1). Here we argue that loss of resistance or increase in conductance, which would be provoked by neural activation, might be of central importance in interpreting the results. Since electrical fields can induce larger changes in transmembrane voltage in resting neurons with low membrane conductance than in active neurons with high conductance, both the immediate effects and the aftereffects of brain stimulation are smaller during voluntary activity compared to the resting condition (Paulus and Rothwell, 2016). The loss of resistance might have also played a role in the other tasks so that any effects of tDCS would have been diminished, especially when the target muscle was involved (i.e., Tasks 1 and 2). Although speculative, the mixed nature of Task 4 that involved both the target and non-target muscles could have a different impact on the interaction between external stimulation and membrane potential and other cellular parameters subject to tDCS. Alternatively, repetitive activation of the target muscle might have had some additional influence that altered a net change in the MEP amplitude, resulting in a tendency toward decreased MEP amplitude after anodal tDCS.

Inter-individual variabilities in the aftereffects of tDCS have been repeatedly reported (López-Alonso et al., 2014; Wiethoff et al., 2014; Chew et al., 2015; Nuzum et al., 2016; Strube et al., 2016). Such variabilities could have resulted in different tDCS effects in combination with different motor tasks, since not all the participants were involved in multiple tasks. Inclusion of “tDCS in rest” condition would have revealed individual responses to tDCS more explicitly and thus could have been used to normalize inter-individual differences. However, we were specifically interested in polarity-specific influence of tDCS and wanted to compare anodal, cathodal, and sham tDCS in combination with the same motor task using a within-subject design. Including another within-subject factor, namely of motor task with four levels, would have resulted in more than 10 sessions for each participant, which we thought was too demanding. It can be an interesting future direction, however, to apply tDCS with a chosen polarity during different motor tasks (including “no task” for the effect of tDCS alone), i.e., using tDCS as a between-subject factor and motor task as a within-subject factor. Such a comparison should further reveal influence of the concurrent motor tasks on tDCS. Monitoring kinematic parameters associated with each task providing immediate feed-back could also provide additional quantitative information to estimate task-related inter-individual variability, which would constitute another study as an interesting future direction. Another measure to be taken in order to reduce source of variability would be a neuronavigation system to make sure that the TMS coil is exactly over the representational area of the target muscle.

A more recent study intriguingly reported a consistent increase in motor cortical excitability after anodal tDCS across four repeated sessions in spite of huge intra-individual variations (Dyke et al., 2016). This observation suggests that there is a fairly good test-retest reproducibility, at least at the group level. The results indicate that the tDCS mode is the factor primarily responsible for the main effects observed in the present study (Figures 2, 3). Therefore, even though it might be difficult to completely explain the mechanistic differences in the interactions of the motor tasks with tDCS, our study design indicates polarity-specific effects of tDCS with regard to a particular motor task.

One of the limitations of this study is that movement parameters, such as force, velocity, and acceleration, were not quantitatively recorded. Such recordings would be useful to infer correlation between the movements and the aftereffects of tDCS. The chance is that some of the movement parameters might turn out to be more important than the type of the movements as a whole in determining the tDCS aftereffects. Also, quantitative measurements of the movement parameters could help us elucidate the source of variability in tDCS aftereffects through a search for correlation between marginal differences in the execution of the task and the tDCS aftereffects, especially on an inter-individual basis. Therefore, it would be an interesting future direction to include quantitative measurements of movement parameters with a similar study design.

In conclusion, the results are congruent with the current notion that the aftereffects of tDCS are highly variable relying on a number of factors including the type of movements executed during stimulation. The implementation of tDCS research or rehabilitation should take such concurrent activities into account to improve the efficacy of tDCS.

## Author contributions

The experiments were planned by AA, WP, and YS. The data was collected by DT and YS. The analysis were done by AA, DT, and YS. All of the authors contributed in writing the manuscript.

### Conflict of interest statement

The authors declare that the research was conducted in the absence of any commercial or financial relationships that could be construed as a potential conflict of interest.

## Supplementary material

Figure S1 Individual time courses of normalized MEP amplitude. Each individual's response is illustrated in a similar way as in Figure 2.

## Abbreviations

ANOVA: analysis of variance
EMG: electromyography
FDI: first dorsal interosseous
M1: primary motor cortex
MEP: motor evoked potential
tDCS: transcranial direct-current stimulation
TMS: transcranial magnetic stimulation.
