# Supplementary figures and images for: Influence of Concurrent Finger Movements on Transcranial Direct Current Stimulation (tDCS)-Induced Aftereffects
Source: Front Behav Neurosci. 2017 Sep 12;11:169. doi: 10.3389/fnbeh.2017.00169 (PMC5600944; doi:10.3389/fnbeh.2017.00169)

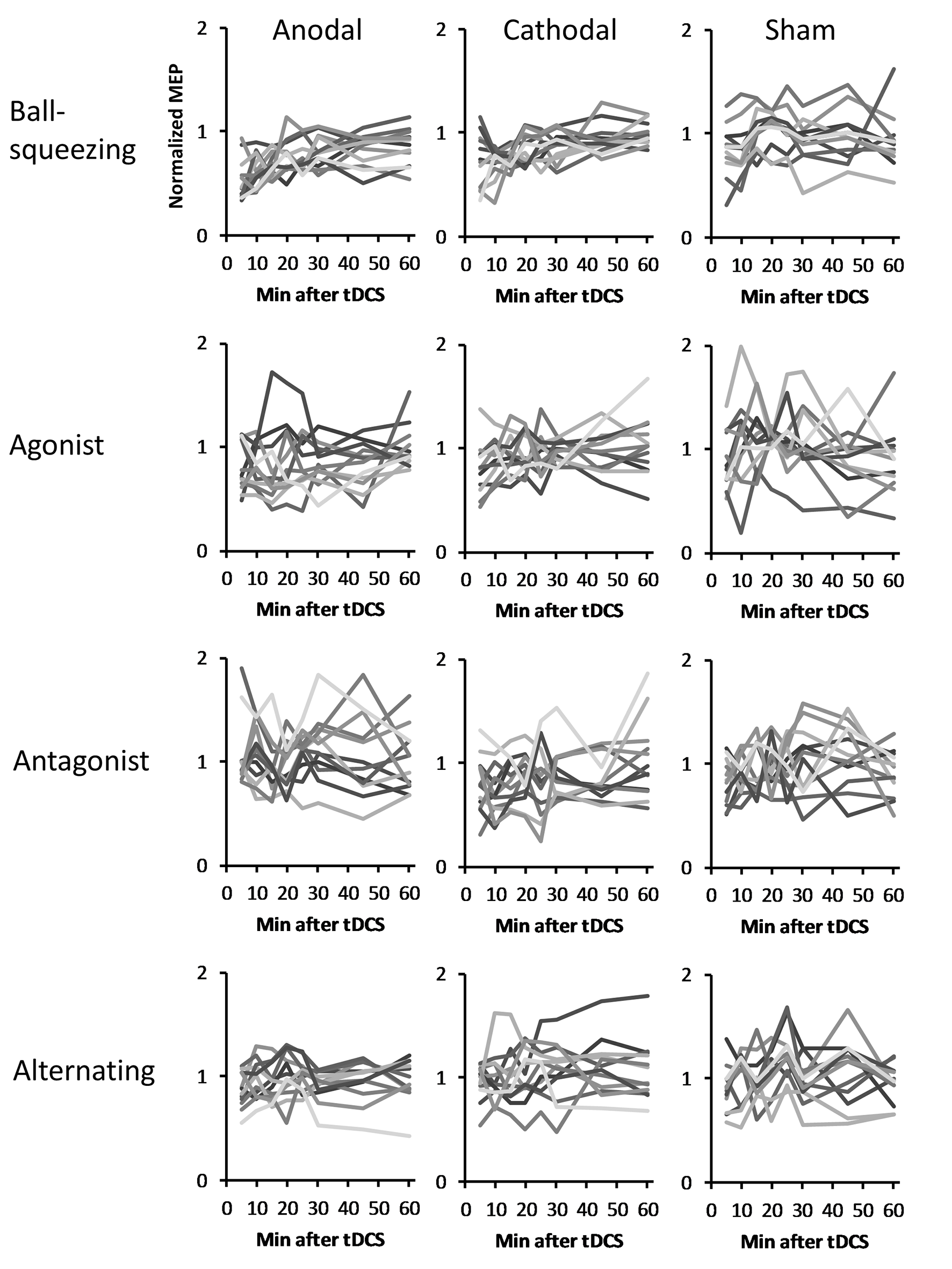

Supplement: Figure S1 — Individual time courses of normalized MEP amplitude. Each individual's response is illustrated in a similar way as in Figure 2. [file Image1.tif]
